# Whole-Genome Sequences of Nine *Francisella* Isolates

**DOI:** 10.1128/genomeA.00941-14

**Published:** 2014-09-25

**Authors:** K. W. Davenport, H. E. Daligault, T. D. Minogue, K. A. Bishop-Lilly, S. M. Broomall, D. C. Bruce, P. S. Chain, S. R. Coyne, K. G. Frey, H. S. Gibbons, J. Jaissle, G. I. Koroleva, J. T. Ladner, G. F. Palacios, C. L. Redden, C. N. Rosenzweig, M. B. Scholz, H. Teshima, S. L. Johnson

**Affiliations:** a Los Alamos National Laboratory Los Alamos, New Mexico, USA; b Diagnostic Systems Division (DSD), United States Army Medical Research Institute of Infectious Diseases (USAMRIID), Fort Detrick, Maryland, USA; c Naval Medical Research Center (NMRC)-Frederick, Fort Detrick, Maryland, USA; d Henry M. Jackson Foundation, Bethesda, Maryland, USA; e Edgewood Chemical Biological Center, Aberdeen Proving Ground, Maryland, USA; f Center for Genome Sciences (CGS), USAMRIID, Fort Detrick, Maryland, USA

## Abstract

Primarily a zoonotic disease, *Francisella tularensis* is a fastidious intracellular pathogen and is listed as a CDC category A pathogen with notably high pathogenicity. Here we present the scaffolded genome assemblies of nine *Francisella* strains: eight *F. tularensis* and one *F. philomiragia*.

## GENOME ANNOUNCEMENT

*Francisella tularensis*, the causative agent of tularemia (rabbit fever) is considered one of the most pathogenic bacterial infections to humans and has been considered a potential biological weapon for at least the last 70 years (1, 2). Members of this group are small nonmotile, capsule-producing, Gram-negative rods and are often difficult to maintain in laboratory culture.

High-quality genomic DNA was extracted from purified isolates of each strain using QIAgen Genome Tip-500 at USARMIID’s Diagnostic Systems Division (DSD). Specifically, 100-mL bacterial cultures were grown to stationary phase and nucleic acid was extracted per the manufacturer’s recommendations. For BSL3 *Francisella*, all extracted material was checked for sterility. If sterility was not achieved, the nucleic acid was passed through a 0.45-µM filter and rechecked for viable organisms before removal from the BSL3 suite.

Sequence data for each draft genome was generated using a combination of Illumina and 454 technologies (3, 4). For each genome, we constructed and sequenced an Illumina library of 100-bp reads at high coverage (224- to 1,205-fold genome coverage) and a separate long-insert paired-end library (Roche 454 Titanium platform, 10- to 68-fold genome coverage). The two datasets were assembled together in Newbler (Roche) and the consensus sequences computationally shredded into 2-Kbp overlapping fake reads (shreds). The raw reads were also assembled in Velvet and those consensus sequences computationally shredded into 1.5-Kbp overlapping shreds (5). Draft data from all platforms were then assembled together with ALLPATHS and the consensus sequences computationally shredded into 10-Kbp overlapping shreds (6). We then integrated the Newbler consensus shreds, Velvet consensus shreds, ALLPATHS consensus shreds, and a subset of the long-insert read-pairs using parallel Phrap (High Performance Software, LLC). Possible misassemblies were corrected and some gap closure was accomplished with manual editing in Consed (7–9).

Automatic annotation for each genome utilized an Ergatis-based workflow at LANL with minor manual curation. Each of the nine scaffolded genomes is available in NCBI (accession numbers listed in Table 1) and the raw data can be provided upon request. Preliminary analysis located the genes encoding *fopA* and *tul4* in all of the *F. tularensis* strains but not in the *F. philomiragia* strain (as expected). In-depth comparative analyses of these and other genomes are currently underway and will be published in subsequent reports.

**TABLE 1:** Basic assembly and annotation statistics

Strain name	Accession no. (no. of contigs)	Genome size (bp) (% G+C content)	No. of CDSs	No. of rRNAs	No. of tRNAs	Isolation source, yr, location	Alternative ID
*F. tularensis* FAC	JOUF00000000 (44)	1,847,973 (32.3)	2,069	7	36	Rabbit, 1984, Illinois, USA	DST6755
*F. tularensis* FAE	JOOR00000000 (9)	1,854,685 (32.3)	2,010	8	36	Rabbit A, 1988, Illinois, USA	DS88-R-147
*F. tularensis* Larsen FAF	JOOS00000000 (2)	1,818,385 (32.3)	1,980	7	36	Unknown, 2006	NIH38
*F. tularensis* *novicida* FAI	JOOT00000000 (25)	1,970,238 (32.3)	1,887	10	38	Water, 2007, Utah, USA	DPG K4C-IS
*F. tularensis* FTU	JOOU00000000 (22)	1,864,194 (32.3)	2,027	7	36	Rabbit, 1988, Illinois, USA	DS87-R-135
*F. tularensis* FTV	JPGP00000000 (96)	1,887,196 (32.2)	2,083	5	34	Rabbit, 1988, Illinois, USA	DS88-R-160
*F. tularensis* FTX	JOOV00000000 (27)	1,880,801 (32.3)	2,053	8	36	Rabbit, 1988, Illinois, USA	DS88-R-144
*F. tularensis* FTZ	JOVO00000000 (19)	1,857,471 (32.3)	5,018	7	36	Rabbit, 1987, Illinois, USA	DS87-R-200
*F. philomiragia* FAJ	JOUE00000000 (6)	2,050,684 (32.6)	1,929	10	39	Water, 2007, Utah, USA	DPG 3B–W

### Nucleotide sequence accession numbers.

Genome accession numbers to public databases are listed in Table 1.
